# Pressure Dependence of the Crystallization Rate for the S-Enantiomer and a Racemic Mixture of Ibuprofen

**DOI:** 10.1021/acs.cgd.1c00980

**Published:** 2021-10-27

**Authors:** Kajetan Koperwas, Wenkang Tu, Frédéric Affouard, Karolina Adrjanowicz, Filip Kaskosz, Marian Paluch

**Affiliations:** † Institute of Physics, University of Silesia in Katowice, 75 Pułku Piechoty 1, 41-500 Chorzów, Poland; ‡ Silesian Center for Education and Interdisciplinary Research SMCEBI, 75 Pułku Piechoty 1a, 41-500 Chorzów, Poland; § Université de Lille, CNRS, INRAE, Centrale Lille, UMR 8207 - UMET - Unité Matériaux et Transformations, F-59000 Lille, France

## Abstract

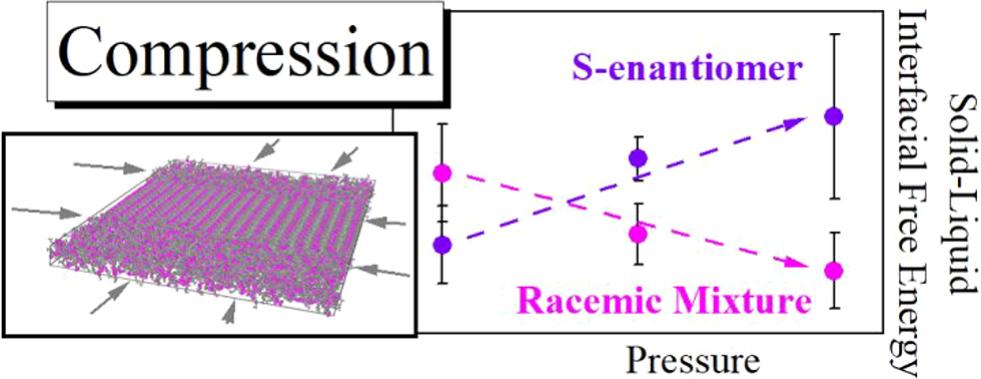

This paper examines the pressure effect on the crystallization rate of the pharmaceutically active enantiomerically pure S-enantiomer and the racemic mixture of the well-known drug ibuprofen. Performed experimental studies revealed that at ambient pressure *S*-ibuprofen crystallizes faster than the racemic mixture. When the pressure increases, the crystallization rate slows down for both systems, but interestingly it is more apparent in the case of the S-enantiomer. It is found that this experimentally observed trend can be understood based on the predictions of the classical nucleation theory. We suggest that the solid–liquid interfacial free energy is the main reason for the observed variations in *S*- and *RS*-ibuprofen’s stability behaviors. Employing a special method of computational studies, i.e., the capillary fluctuation method, we show that the increase in pressure affects the solid–liquid interfacial free energy for *S*- and *RS*-ibuprofen in an entirely different way. Importantly, the detected differences correspond to the experimentally observed variations in the overall crystallization rates.

## Abstract 2

The pure S-enantiomer and racemic mixture of ibuprofen are studied in the context of the crystallization rate under compression. The experimentally observed slowing down in the crystallization rate is more apparent for the pure S-enantiomer, which is ascribed to the different pressure behavior of the solid−liquid interfacial free energy.

## Introduction

Through the last few decades, the rising interest in pharmaceuticals prepared in the amorphous state has been observed. It is mainly due to the higher dissolving rate of this physical form in contrast to the typical crystalline phase, which translates into the higher bioavailability of the amorphous drug.^1−3^ However, all merits of the amorphous pharmaceutical may be lost during its storage because the sample might crystallize. Since the amorphous state is thermodynamically unstable, it implies a possible rapid transformation of the system to the crystalline form, which is thermodynamically stable. Therefore, the effective preparation of the amorphous drugs would be possible only when such undesired events are limited.

The first and necessary step in this challenging task is a detailed understanding of the crystallization process. One of the most popular and widely used ways to describe the crystallization process is the classical nucleation theory (CNT), of which a remarkable advantage is simplicity.^4−6^ According to this concept, the crystallization process is divided into two steps, i.e., nucleation and crystal growth. The first is characterized by the nucleation rate,^7,8^*N*, which parametrizes the number of crystalline nuclei created within a given space and time. Subsequently, the formed nucleus which has reached a certain critical size grows. The crystal growth rate, *U*, describes the velocity of the increase of the crystal order within the liquid. *N* and *U* are two separate processes, but they actually depend on the same physical properties: systems’ dynamics, i.e., the diffusivity of the molecules at liquid phase, *D*, and thermodynamic features characterized by the driving force for the crystallization Δ*G*, which is the difference between the Gibbs free energy of liquid and crystal.^9−12^ The above physical quantities are easily experimentally obtained. However, according to the CNT, the nucleation strongly depends on the solid–liquid interfacial free energy, γ, which is the reversible work required to create a unit area of the interface.^13^ γ can be uniquely defined if the interface between the two phases is planar and the chemical potentials of the above phases are identical. These conditions are fulfilled for the liquid–vapor interface at equilibrium for which extensive experimental examinations have been performed.^14−16^ However, for solid–liquid interfaces, the plane of the interface depends on the crystallographic orientation.^17,18^ The latter implies that, in general, γ is an anisotropic quantity, and therefore its direct experimental examination is a challenging task.^18,19^

The fact that γ depends on the crystal morphology^20^ also has important consequences for preparing the amorphous drugs. Considering the pharmaceuticals’ stability behavior, one should take into account that those materials frequently occur in various enantiomeric forms, which crystallize to the different primitive cells. Hence, the almost identical molecules of two enantiomers might exhibit various crystallization tendencies.^21^ Interestingly, the dynamics of the enantiomeric systems does not strongly differ, and therefore their effect on the crystallization process might be excluded.^22^ Consequently, enantiomers and their mixture seem to be interesting candidates to examine the role of thermodynamics on the crystallization process. One of the popular and commonly used examples of a pharmaceutical existing in two enantiomeric forms is ibuprofen. Conveniently, it is produced as a racemic mixture, whereas only the S-enantiomer is therapeutically effective. Importantly, the crystal structures of separated enantiomers and their racemic mixture differ.^23^ Hence, one might expect differences in their γ and Δ*G* values, which would lead to various stability behaviors.

Taking the above into account, in this paper, we examined the crystallization tendency for the S-enantiomer of ibuprofen ((*S*)-(+)-2-[4-isobutylphenyl] propionic acid) and its racemic mixture ((2*RS*)-2-[4-(2-methylpropyl) phenyl] propanoic acid). Experimental studies enabled a detailed comparison of the overall crystallization rates for two studied materials, at ambient and elevated pressures. Subsequently, we used the CNT to disclose the reason for the observed differences. Our theoretical analysis and computational calculations of the interfacial free energy revealed that during the increase in the pressure, the solid–liquid interfacial free energy at melting conditions for *RS*-ibuprofen decreases, whereas for *S*-ibuprofen, it increases. Interestingly, the effect of the compression on Δ*G* is similar for both examined materials. Consequently, our findings suggest that the thermodynamic aspects of the crystallization process are prominently sensitive for a final crystal structure which is formed as well as, hypothetically, on the composition of the liquid sample.

## Experimental Section

### Materials

Ibuprofen (being a racemic mixture of (*S*)-ibuprofen and (*R*)-ibuprofen and is labeled as *RS*-ibuprofen in the context) of a purity greater than 98% was purchased from Hubei Biocause Pharmaceutical Co. Ltd., while (*S*)-ibuprofen (labeled as *S*-ibuprofen in the context) of 99% purity was purchased from Merck. For details on chemical structures of the R and S enantiomers, readers can refer to the earlier literature.^24^ The samples were used without further purification.

### Methods

#### Standard Differential Scanning Calorimetry (DSC) Measurements

Thermodynamic properties of the tested materials were investigated by using a Mettler-Toledo DSC apparatus equipped with a liquid nitrogen cooling accessory and an HSS8 ceramic sensor (a heat flux sensor with 120 thermocouples). Indium and zinc standards were used for the temperature and enthalpy calibrations. Samples with a mass of about 15 mg were placed in aluminum crucibles and sealed. Experiments were performed within temperature ranges of 200–363 K and 200–343 K for *RS*-ibuprofen and *S*-ibuprofen, respectively, with fixed heating/cooling rates of 10 K/min used during the heating–cooling-reheating cycles. At least three DSC runs following the same protocol were performed for the studied samples to ensure data reproducibility. To be consistent with the literature results,^25^ in this work the melting point, *T*_m_, is also determined as the onset temperature.

#### Temperature-Modulated Differential Scanning Calorimetry (TMDSC) Measurements

In order to study the heat capacity changes, Δ*C*_*p*_, of the tested samples in the glass transition region, we applied the stochastic temperature–modulated differential scanning calorimetry (TMDSC) technique implemented by Mettler-Toledo TOPEM. The quenched *S*-ibuprofen and *RS*-ibuprofen samples were measured in the same temperature region of 200–267 K at a heating rate of 1 K/min. In the experiments, a temperature amplitude of 0.5 K for the pulses was selected at a switching time range with minimum and maximum values of 15 and 30 s, respectively. As a result, we can obtain the heat capacity *C*_*p*_ curves, from which the glass transition temperature, *T*_g_ (namely, the point corresponding to the midpoint inflection of the extrapolated onset and end of the *C*_*p*_ curve) as well as the Δ*C*_*p*_ value at this temperature can be determined.

#### Broadband Dielectric Spectroscopy

Dielectric measurements at ambient pressure were performed in the frequency range of 10^–2^ to 10^6^ Hz using a Novocontrol Alpha analyzer. The Quattro system together with a nitrogen gas cryostat was employed to control the temperature, which ensures a temperature stability within ±0.1 K. The investigated materials were placed in between two stainless steel electrodes (20 mm diameter) with a gap generated by using the Kapton spacer of ∼0.05 mm thickness. As an initial step, *S*- and *RS*-ibuprofen were, respectively, kept at temperatures of *T* = 343 K and *T* = 363 K for 15 min to ensure the complete melting. Subsequently, the samples were cooled to 203 K to enter the glassy states at a rate of ∼10 K/min. After this, the dielectric responses in the representations of the real (ε′) and imaginary (ε″) parts of complex permittivity (ε*) were recorded upon slowly heating the amorphous samples (0.5 K/min) to high temperatures. Besides the non-isothermal studies, the dielectric technique was used to perform the isothermal crystallization studies. Noted is that the samples were heated up to the desired crystallization temperature in the supercooled liquid state immediately after achieving the glassy state at *T* = 203 K via rapid quenching. In addition, to avoid the possible degradation of the tested chemicals, a new sample was prepared for each crystallization experiment.

In the case of pressure-dependent dielectric studies, a high-pressure system built by Unipress (Institute of High-Pressure Physics, Warsaw, Poland) was used. The high-pressure setup consists of an MP5 micropump, an MVX-30 vessel, and a control unit, which allows regulation of the pressure within a precision of 1 MPa. The pressure was exerted by using silicon oil transmitted to the pressure chamber through a system of capillary tubes (Novo Swiss). The temperature inside the pressure vessel was controlled using a highly dynamic temperature control system (Presto W85, Julabo). Dielectric measurements were carried out with a capacitor of the same geometry as that used for ambient pressure measurements (diameter 20 mm, gap 0.05 mm, Kapton spacer). Prior to the experiments, the capacitor was sealed with Teflon tape and then placed inside the pressure chamber filled with pressure transmitting silicon oil. In this work, molecular dynamics of *S*-ibuprofen were investigated in both isothermal and isobaric experiments. Moreover, crystallization kinetics of *S*-ibuprofen and *RS*-ibuprofen was compared under both ambient and elevated pressures. In each crystallization experiment, after completing the liquid-crystal transformation, the temperature was increased slowly (0.5 K/min) to melt the samples, and the melting points of the studied samples were determined from the temperature-dependent ε′ evolutions.

#### Molecular Dynamics Simulation

The standard molecular dynamics simulations have been carried out using the GROMACS software^26−29^ at conditions of the constant temperature and pressure provided by the Nose-Hoover thermostat^30,31^ and the Martyna–Tuckerman–Tobias–Klein barostat.^32,33^ The interactions between nonbonded and bonded atoms are defined by the OPLSAA force field.^34^ The nonbonded interactions are cutoff at a distance equal to 1 nm, whereas the used time step equals 0.001 ps. The crystal structures for *S*- and *RS*-ibuprofen were constructed according refs (35 and 36), respectively. The crystal and liquid simulation boxes, which consist of 2302 molecules for the S-enantiomer and 2304 molecules for the racemic mixture, were heated or cooled from experimentally determined melting temperatures up to a temperature higher or lower for at least 120 K. The difference in the temperature between subsequent simulation runs was equal to 10 K. Each simulation run lasts for 1.5 ns during which the first 0.5 ns was dedicated for the equilibration of the system, whereas the collected data during the last 1.0 ns were used to estimate the crystal and liquid densities, enthalpies, and diffusion constant for the liquid phase. The latter have been done on the basis of the mean-square displacement using the GROMACS software.

## Results

**Figure 1:**
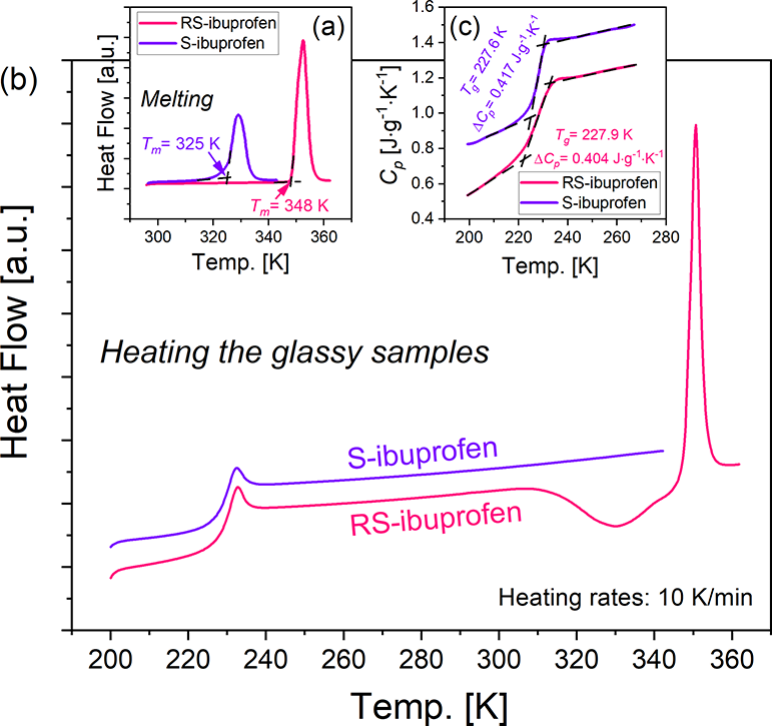
DSC traces for *S*-ibuprofen and *RS*-ibuprofen were recorded upon heating (10 K/min) the samples in the crystalline (a) and glassy (b) states. Panel (c): Heat capacity *C*_*p*_ curves for both chemicals obtained within the TOPEM measurements. The temperature range is from 200 to 267 K, and the heating rate is 1 K/min.

The thermal behaviors of *S*-ibuprofen and *RS*-ibuprofen were investigated using the standard DSC and TMDSC techniques. In Figure 1a, for both studied materials, we can observe pronounced endothermic peaks signifying the melting processes. The melting points, *T*_m_’s, were determined as 325 and 348 K, respectively, for *S*-ibuprofen and *RS*-ibuprofen. Noteworthily, Rietveld et al. reviewed numerous studies and reported that the melting points of *S*-ibuprofen and *RS*-ibuprofen have averages of 323 ± 4 K and 349 ± 2 K, respectively.^37^ Thus, the melting temperatures determined in this work agree well with the literature data. To ensure the complete melting of the samples, *S*-ibuprofen and *RS*-ibuprofen were, respectively, kept at *T* = 343 K and *T* = 363 K for 5 min. Then, the samples were quenched to 200 K at a rate of 10 K/min to enter their glassy states. In Figure 1b, DSC thermograms recorded upon heating the glassy samples at 10 K/min are revealed. We can see that the glass transition phenomena of both samples occurred in the same temperature region. Upon further heating to temperatures above *T* = 300 K, an exothermic peak (cold crystallization) immediately followed by an endothermic peak (melting process) is observed for *RS*-ibuprofen. Nevertheless, such peaks are not visible for *S*-ibuprofen. As a next step, we used TMDSC to study the heat capacity *C*_*p*_ changes of both samples within the glass transition region. Immediately after the melted samples were quenched to enter the glassy states, the temperature was slowly increased (1 K/min) from 200 to 267 K. On the basis of the *C*_*p*_ curves ascertained upon heating the samples (as seen in Figure 1c), we can determine approximately the same *T*_g_ and Δ*C*_*p*_ values, which are 227.6 K and 0.417 J·g^–1^·K^–1^ for *S*-ibuprofen and 227.9 K and 0.404 J·g^–1^·K^–1^ for *RS*-ibuprofen. The thermodynamic parameters, namely, *T*_g_, Δ*C*_*p*_ and *T*_m_, are compiled in Table 1.

**Table 1:** Glass Transition and Melting Parameters Determined for *RS*-Ibuprofen and *S*-Ibuprofen at Ambient Pressure by Using the Techniques of Differential Scanning Calorimetry (DSC) and Dielectric Spectroscopy (DS) and Melting Point Data Obtained at a High Pressure of 200 MPa by Using the DS

	DSC			DS			
materials	*T*_g_ (K)	Δ*C*_*p*_ (J·g^–1^·K^–1^)	*T*_m_ (K)	*T*_g_, *p* = 0.1 MPa (K)	*m*, *p* = 0.1 MPa	*T*_m_, *p* = 0.1 MPa (K)	*T*_m_, *p* = 200 MPa (K)
*RS*-ibuprofen	227.9	0.404	348	224	87	345	399.1
*S*-ibuprofen	227.6	0.417	325	225	82.2	321.5	374.5

**Figure 2:**
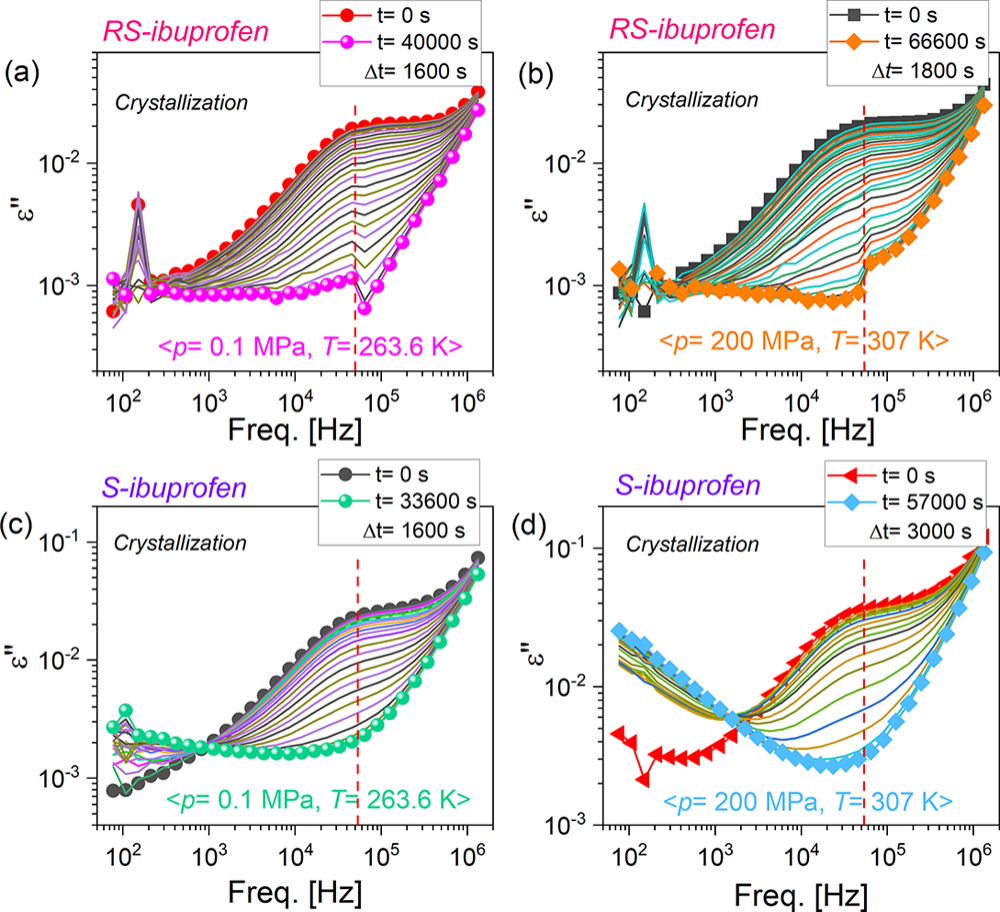
Time evolutions of the dielectric loss ε″ spectra for *RS*-ibuprofen (a and b) and *S*-ibuprofen (c and d) during the isothermal crystallization measurements. Panels (a) and (c) show the results for *RS*-ibuprofen and *S*-ibuprofen recorded under the same conditions of *p* = 0.1 MPa and *T* = 263.6 K, respectively. Panels (b) and (d) depict the results for *RS*-ibuprofen and *S*-ibuprofen recorded under the same condition of *p* = 200 MPa and *T* = 307 K, respectively. In each panel, spectra obtained at the beginning and end of the crystallization process are marked.

**Figure 3:**
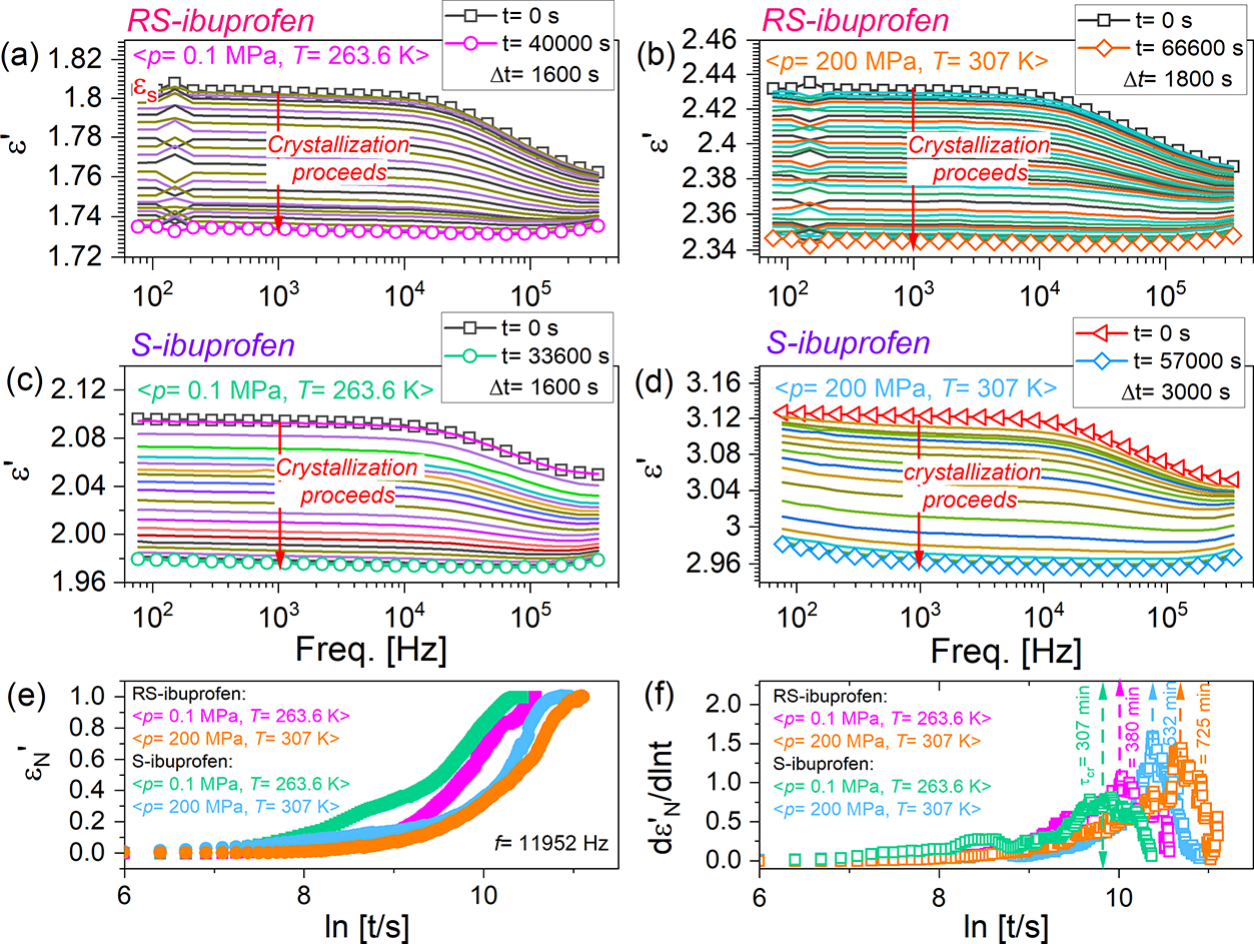
Time evolutions of the dielectric ε′ spectra for *RS*-ibuprofen (a and b) and *S*-ibuprofen (c and d) during the isothermal crystallization measurements. Results obtained for *RS*-ibuprofen and *S*-ibuprofen under the same condition of *p* = 0.1 MPa, *T* = 263.6 K are depicted in panels (a) and (c), respectively. Panels (b) and (d) show the results for *RS*-ibuprofen and *S*-ibuprofen recorded under the same conditions of *p* = 200 MPa, *T* = 307 K. Panel (e): relations of ε_*N*_^′^–ln *t* for the tested *S*-ibuprofen and *RS*-ibuprofen samples. The results are ascertained at a fixed frequency of 11 952 Hz. Panel (f): time dependences of the first derivative of ε_*N*_^′^ against the natural logarithm of time (d*ε*_*N*_^′^/d ln *t*) in terms of the Avramov approach.

As a matter of fact, we also performed dielectric studies on the glass transition behaviors of *S*-ibuprofen under both ambient and elevated pressures (see Figures S1). When comparing the ascertained results to the data for *RS*-ibuprofen as reported in our previous work,^38^ we noticed the identical glass transition dynamics for *S*-ibuprofen and *RS*-ibuprofen (see Figure S2) at various pressure conditions. Nonetheless, it is of interest to make clear whether the crystallization kinetics of these two materials will be different under both ambient and elevated pressures. Hence, we applied the dielectric technique to perform the isothermal crystallization measurements for *S*-ibuprofen and *RS*-ibuprofen under two kinds of conditions, namely, *p* = 0.1 MPa, *T* = 263.6 K and *p* = 200 MPa, *T* = 307 K, where the tested samples exhibit the same molecular mobility. As demonstrated in each panel of Figure 2, the initial dielectric loss ε″ spectra are characterized by the structural α-relaxation peaks located at the same frequency (∼5.5 × 10^4^ Hz). Time evolutions of the dielectric ε″ and ε′ spectra are compiled in Figures 2 and 3, respectively. The kinks, which might be observed in Figure 2a,b, are the experimental artifacts, resulting probably due to the complexity of a performed high-pressure experiment. As crystallization proceeds, the α-peak in each panel of Figure 2 shows a gradually decreased intensity that ultimately disappears. In the case of the dielectric ε′ spectra (see Figure 3a–d), the crystallization phenomenon is reflected by the decreased static permittivity ε_s_. At the ultimate stage of crystallization, ε_s_ ceases to change with time. For analyzing the crystallization process, we used a normalized parameter, ε_*N*_^′^, which can be expressed as
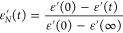
1where ε′(0) and ε′(∞) are the values of static permittivity at the initial and ultimate stages of crystallization, and ε′(*t*) is the value at time *t*. In Figure 3e, we prepared plots of ε_*N*_^′^ versus the natural logarithm of time ln(*t*) for the tested four samples. Moreover, in Figure 3f, the first derivative of ε_*N*_^′^ against the natural logarithm of time (d*ε*_*N*_^′^/d ln(*t*)) is plotted as functions of ln *t* in terms of the so-called Avramov approach. From each d*ε*_*N*_^′^/d ln(*t*) - ln(*t*) relation in Figure 3f, we can determine a critical parameter (τ_cr_), which represents the characteristic time for the overall crystallization process. For *S*-ibuprofen and *RS*-ibuprofen samples held at *p* = 0.1 MPa, *T* = 263.6 K, the τ_cr_ values are 307 and 380 min, respectively. When the measurements were carried out at *p* = 200 MPa, *T* = 307 K, the τ_cr_ values for *S*-ibuprofen and *RS*-ibuprofen are 532 and 725 min, respectively. Apparently, both *S*-ibuprofen and *RS*-ibuprofen exhibit slower crystallization kinetics at high pressure with respect to the ambient pressure conditions. A similar phenomenon that pressure can suppress the crystallization has also been observed for R,S-racemic mixture of ketoprofen.^39^ Additionally, as compared to *RS*-ibuprofen, faster crystallization occurred for *S*-ibuprofen under both ambient and elevated pressures.

**Figure 4:**
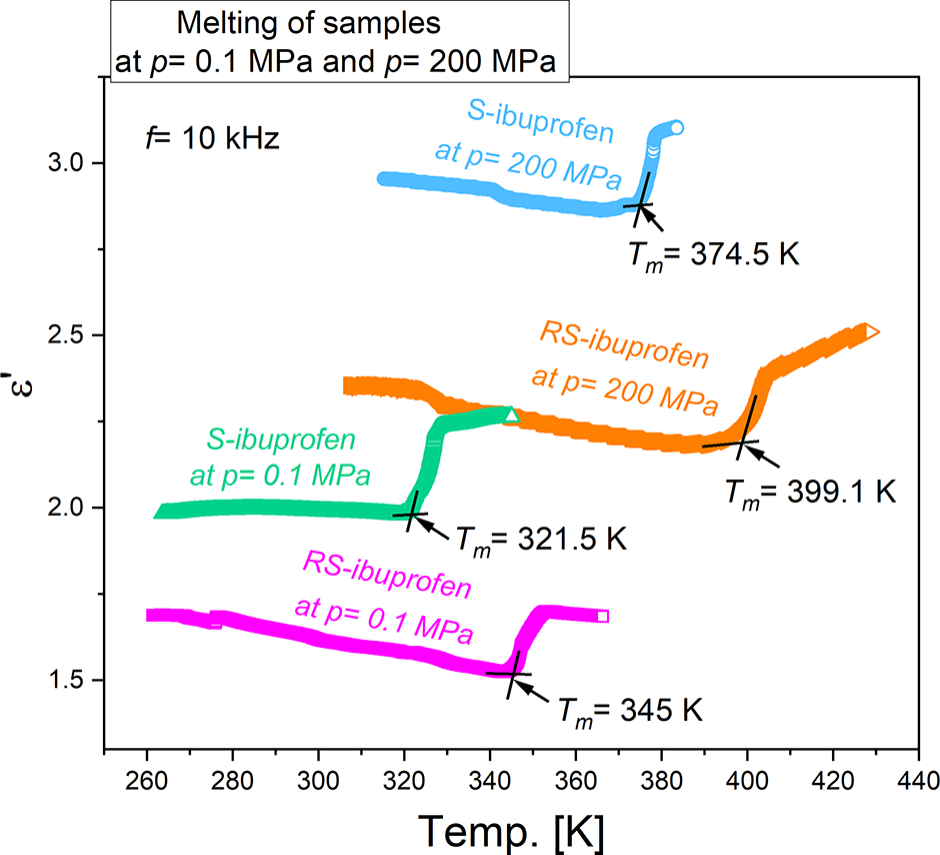
Temperature dependences of ε′ were recorded at a fixed frequency of 10 kHz upon heating the studied *S*-ibuprofen and *RS*-ibuprofen samples after crystallization measurements were completed at pressures of 0.1 and 200 MPa. The melting points as determined by the onset temperatures of the step increases of ε′ are given.

As a next step, immediately after the isothermal crystallization measurements, the samples were heated up slowly with 0.5 K/min for the determination of melting points under the experimental conditions. Figure 4 depicts the temperature dependences of ε′ recorded at a fixed frequency of 10 kHz upon heating the studied crystalline *S*-ibuprofen and *RS*-ibuprofen samples. In each curve, the melting process is displayed as the step increase of ε′, and the onset temperature is defined as the melting point. As we see, for *S*-ibuprofen and *RS*-ibuprofen, *T*_m_ values are, respectively, 321.5 and 345 K at *p* = 0.1 MPa and 374.5 and 399.1 K at *p* = 200 MPa. The results at ambient pressure are in good agreement with the aforementioned DSC results (see Table 1). In addition, the same *T*_m_ value of 345 K has also been reported by Brás et al. in the dielectric studies of *RS*-ibuprofen at ambient pressure.^25^

**Figure 5:**
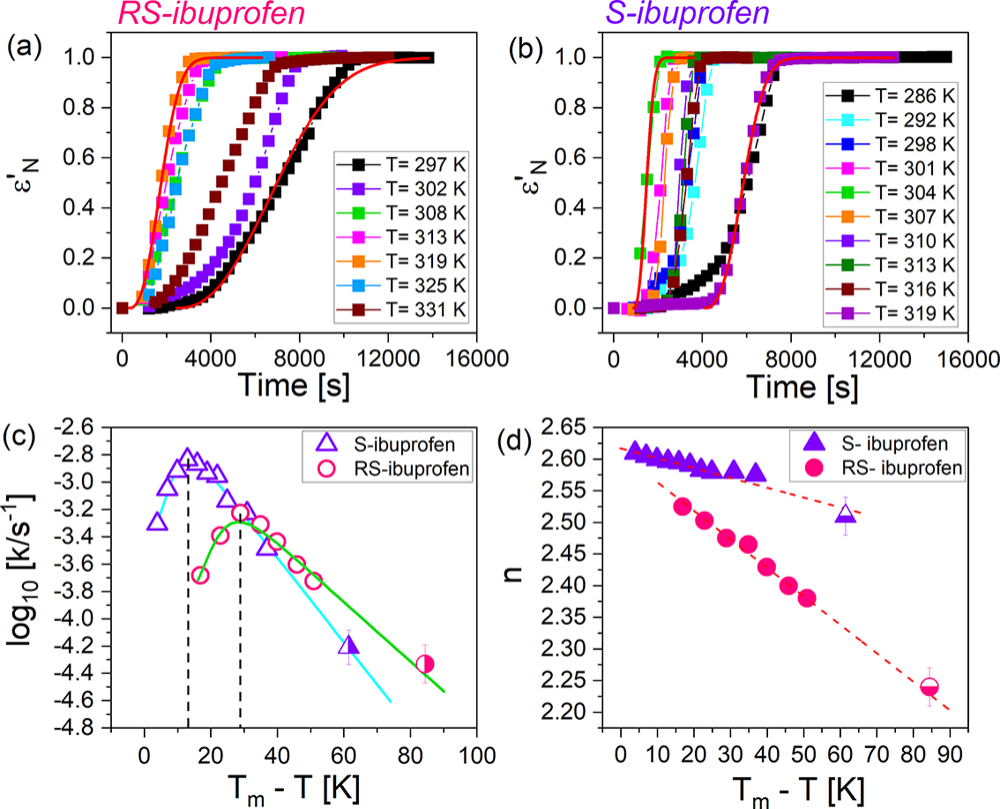
Relations of ε_*N*_^′^–*T* ascertained from isothermal crystallization measurements for *RS*-ibuprofen (a) and *S*-ibuprofen (b) at various temperatures under ambient pressure. Red solid lines represent the fitting results in terms of the Avrami equation. For both tested samples, evolutions of the obtained kinetic parameters *k* (c) and *n* (d) along with the degree of supercooling are shown. In panels (c) and (d), the right-half and bottom-half filled symbols represent *k* and *n* values for both tested samples, which were measured using the high-pressure setup under the ambient pressure (as shown in Figures 2 and 3). In panel (c), solid lines represent the fits to the experimental data with the use of an exponential function plus a linear term, and the dashed vertical lines reveal the positions of the maximum crystallization rates. In panel (d), red dashed lines exhibit the evolution tendency of the *n* values.

In order to gain a more thorough picture of crystallization kinetics of *S*- and *RS*-ibuprofen, we conducted isothermal measurements for each chemical at various temperatures under ambient pressure. Figure 5a,b depict the corresponding ε_*N*_^′^–*T* relations ascertained for *RS*-ibuprofen and *S*-ibuprofen, respectively. In order to analyze the data, we applied the Avrami equation, which can be expressed as follows,^40^
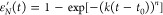
2where *k* and *n* are the overall crystallization rate constant and Avrami exponent, respectively. *t*_0_ denotes the incubation time, which represents a time required to produce crystal nuclei of sufficient size for further growth. To avoid crowding, only the exemplified fitting results at selected temperatures are shown in Figure 5a,b. As a next step, we prepared the plots of determined *k* and *n* values as functions of the degree of supercooling (Δ*T* = *T*_m_ – *T*). Here, the *T*_m_ values of 348 K for *RS*-ibuprofen and 325 K for *S*-ibuprofen were used. In Figure 5c, arch-shaped *k*–Δ*T* correlations are observed for both tested materials. The maximum crystallization rate of *S*-ibuprofen occurs at Δ*T* ≈ 15 K while that of *RS*-ibuprofen occurs at Δ*T* ≈ 29 K. Slower crystallization kinetics for *RS*-ibuprofen than *S*-ibuprofen is noticed at the same Δ*T* below 32 K. Nonetheless, the crystallization rate of *RS*-ibuprofen slightly exceeds that of *S*-ibuprofen at the same Δ*T* above 32 K. In Figure 5d, the evolutions of the Avrami exponent *n* along with the temperature are shown. For both samples, a gradually decreasing trend of *n* with increasing Δ*T* is noticed, and *n* values between 2 and 3 indicate a thermal nucleation followed by two-dimensional crystal growth.^41^

## Discussion

The determination of the reasons that *S*-ibuprofen crystallizes faster than the racemic mixture can be made if one employs the theoretical description of the considered process. As we have already mentioned, the most popular approach for a description of the crystallization process is the CNT. According to this concept, in the most basic and theoretically fundamental case, i.e., for three-dimensional crystal growth, the overall crystallization rate follows the equation below:
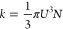
3The nucleation rate *N* can be estimated according to the formula
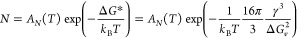
4where *A*_*N*_(*T*) is a kinetic prefactor, while ΔG* is the nucleation barrier. The kinetic prefactor can be rewritten as *A*_*N*_(*T*) = 24 *Zρ*_*l*_*Dn*^2/3^/λ^2^, where the Zeldovich parameter *Z* = (Δ*G*_v_/6*πk*_B_*Tnρ*_c_)^1/2^, the number of molecules in the critical nucleus *n* = (4/3)(*πr*^3^ρ_c_) of the radius *r* = 2γ/Δ*G*_*v*_, the atomic jump distance λ = (1/ρ_l_)^1/3^, whereas ρ_l_ and ρ_c_ are the number density of liquid and crystal, respectively. Δ*G*_v_ is the driving force per volume unit which can be obtained from Δ*G* and ρ_c_. The next physical quantity influencing the overall crystallization is the crystal growth, the role of which can be computed by the following expression
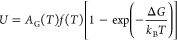
5where *A*_G_(*T*) ≈ *Dρ*_c_^–1/3^ρ_l_^2/3^ and describes the molecular mobility, whereas *f*(*T*) parametrizes the growth mechanism. As it was suggested for other real material, the normal growth model, which assumes that *f* is constant and equal to 1, exhibits the best accuracy with the experimental results so far.^42^ As a consequence, to use eqs 4 and 5, knowledge of the temperature dependence of volume for liquid and crystal phases, *D*(*T*), Δ*G*(*T*), as well as γ(*T*) is required. The values of the first three quantities can be obtained from the standard experiments. However, the direct experimental measure of γ is a challenging task even for pure material.^19,43,44^ Moreover, the experimentally obtained values strongly depend on the used technique. Fortunately, the alternative ways to calculate the value of γ are derived by the computational experiments. Recently, two main approaches have been suggested: the cleaving potential method^45−47^ and the capillary fluctuation method.^17,48−51^ In the cleaving potential method, the biphasic solid–liquid system is transformed into two separate systems (liquid and solid) by use of the external potentials. Then measurement of the work made by those potentials during the transformation process enables estimation of γ. It should be mentioned that precise application of this method requires precise control of the transformation process to ensure its reversibly, which implies some technical difficulties.^52^ The employment of capillary fluctuation method (CFM) can be done in a more direct way because it requires only one simulation of a biphasic system during which the fluctuations of the interfaces are measured. The parametrized stiffness of the interface can be related to the γ. Briefly speaking, the cleaving method is considered as one of more precision, whereas the CFM is characterized by higher sensitivity to the anisotropy in γ. The discussed methods have been used to estimate γ values for model systems such as hard-spheres^46,53^ and Lennard-Jones.^47,50,54^ However, for realistic systems, the CFM is more often employed, which is probably because CFM requires only one simulation run, and any knowledge of the complex process of creating interface from separated bulk systems is not needed. Consequently, using the CFM, the γ values can be estimated for metallic compounds,^17,18,48,49^ alloys,^55,56^ and few molecular systems^57^ including pharmaceuticals.^20,58^ Hence, we decided to apply this method for studying the differences in γ, nucleation rate, and an overall crystallization tendency between the S-enantiomer and the racemic mixture of ibuprofen.

**Figure 6:**
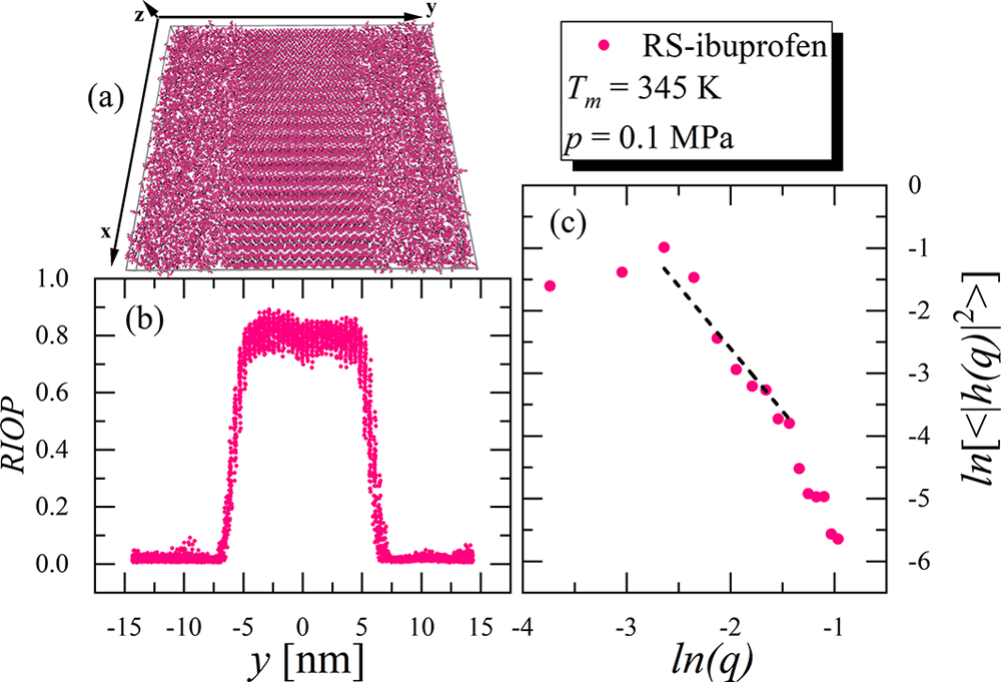
In panel (a) the snapshot of the simulation run leading to estimation of the IFE is shown. The two solid–liquid interfaces positioned along the *x* axis are distinguished. The simulation box size along the *z* axis is significantly smaller than along the *x* and *y* axis to provide the quasi one-dimensional solid–liquid interface. The rotational-invariant order parameter calculated along the direction perpendicular to the solid–liquid interface is presented in panel (b). Panel (c) shows the fluctuation spectrum of the interface height. The dashed line is a fit of the linear function with the slope equal to −2.

At this point, it has to be noted that CFM (as well as cleaving potential method) can be applied only at the melting conditions, i.e., at the conditions at which the crystal and liquid coexist. Thus, we need to confirm that the experimentally determined *T*_m_ is valid also for simulated systems. The latter can be done by the examination of the biphasic system stability. In this order, we equilibrated the crystal structure at *T*_m_ and subsequently melted it at a sufficiently high temperature. During this step, the conditions of constant temperature and volume (NVT) were kept. This procedure enables us to obtain the identical boxes filled by the crystal and liquid. However, to ensure the identical pressure conditions, the liquid box was elongated providing the density equal to the liquid density at the studied melting conditions estimated from the independent simulation run. Subsequently, the liquid system was simulated at NVT conditions in order to fill the box completely. In the described way, the crystal box and the elongated box possessed an identical plane, which enabled its joint (the small gap between boxes has been ensured to provide the overlapping of the atoms belonging to different phases). During the NPT simulation, which last for 10 ns, any melting or crystallization event has not been observed, confirming the utility of experimentally determined melting temperatures for further computational experiments including one determining the IFE by CFM. The final configuration of the simulated biphasic system consisting of *RS*-ibuprofen molecules is presented in Supporting Information (Figure S3). At this point, it must be noted that the use of CFM requires construction of the quasi-one-dimensional interfaces. Therefore, to ensure the required geometrical condition, a special biphasic simulation box must be created; i.e., when created interface is parallel to the length of the system, *L*_*x*_, its thickness must be much smaller than its width, *L*_*z*_ ≪ *L*_*y*_. The snapshot of the simulation box created in the described way is presented in Figure 6a. At this point, it must be noted that application of the periodic boundary to the simulation implies the existence of two interfaces, which during the simulation run fluctuate only in the one dimension (*y*). The temporary position of the interfaces can be estimated by the calculation of the rotational-invariant order parameter^18,59−63^ (RIOP) for geometrical center of molecules. The latter enables the distinction between solid-like and liquid-like molecules because the solid-like molecules are characterized by significantly higher values of the order parameter. The example of obtained result for *RS*-ibuprofen is presented in Figure 6b, where the calculated RIOP for each molecule are plotted as a function of the position of the molecule in the dimension perpendicular to the interface plane (*y* direction).

**Figure 7:**
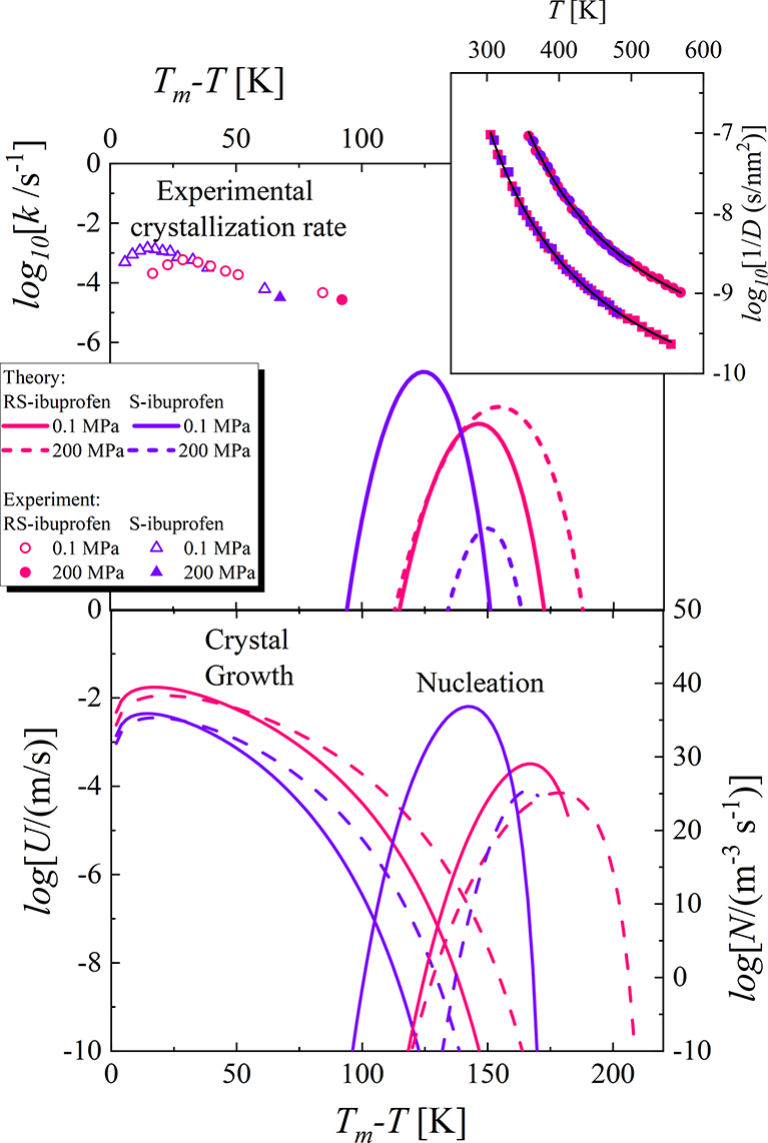
The inset presents the diffusion constant for *S*- and *RS*-ibuprofen at ambient and elevated pressure. The solid lines represent the fit of the VFT equation. In the upper main panel, the solid and dashed lines represent the overall crystallization rate predicted by the combination of CNT and the normal model of growth at ambient and elevated pressure. Results of an experimental examination of *S*- and *RS*-ibuprofen are presented as points. In the lower panel, the solid and dashed curves represent the theoretical predictions for the crystal growth and nucleation rates, respectively.

The solid-like and liquid-like can be clearly distinct and the evolution of the RIOP can be described by the following function , where *o*_s,l_ are the average values of the RIOP in the solid and liquid, δ_1,2_ are effective widths of the interfaces, and *h*_1,2_(*x*) are a functions describing the positions of the interfaces in capillaries, i.e., sections from *x* to *x* + Δ, which are orthogonal to the interface. During the simulation run, the *h*(*x*) describes the interface fluctuations. The latter can be Fourier-transformed leading to the expression for power spectrum of the quasi-one-dimensional interface
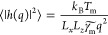
6where *h*(*q*) is the one-dimensional Fourier transform of *h*(*x*) with *q* as the wavenumber, ⟨ ⟩ denotes the time average, *k*_B_ is the Boltzmann constant, and *L*_*x*_ and *L*_*z*_ are width and thickness of the simulation box. According to the CFM the interfacial stiffness, , is used as a fair estimation of the γ_m_. At this point, it is worth mentioning that different orientations of the crystal structure enable determination of anisotropy of γ_m_. However, the direct studies of model^48−50,64^ and realistic^52,57,65,66^ systems suggest that this effect is usually relatively weak, and therefore γ_m_ can be obtained from  determined from a single crystallographic orientation. Then at small *q*, where eq 6 is valid,^48^ ln(⟨|*h*(*q*)|^2^⟩) is a linear function of ln(*q*) with a slope equals −2 and intercept which is directly related to . Hence γ_m_ can be calculated by fitting the obtained dependence of ⟨|*h*(*q*)|^2^⟩ on *q* (expressed in logarithmic scales) to the linear function with the constant slope equal to −2 and analyzing its intercept; see Figure 6c, where the discussed fit is presented for *RS*-ibuprofen. At ambient pressure, the obtained values of γ_m_ equal 51.1 and 67.0 mJ/m^2^ for *S*- and *RS*-ibuprofen, respectively. Subsequently, the temperature dependence of IFE was predicted by the equation inspired by the Turnbull law^67^
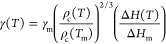
7where Δ*H*(*T*) is the enthalpy difference between the liquid and the crystal. *H*(*T*) for both phases were obtained directly from the computer simulation during cooling of the liquid and heating of the crystal. Both cooling and heating have been performed at conditions of constant temperature and pressure. At each thermodynamic condition, the system remained for 1.5 ns (the first 0.5 ns was spared for the equilibration of the system, whereas the data were collected in the next 1.0 ns). After that, the temperature was changed by 10 K. Additionally, from those computational experiments, we determined the ρ(*T*) for crystal and liquid phases for both *S*- and *RS*-ibuprofen. Simultaneously, using the well-known thermodynamic relation, d*S* = d*H*/*T* (enthalpy was obtained directly from the simulation) and based on the standard integration method, Δ*G* = −∫_*T*_m__^*T*^Δ*S* d*T* (Δ*S* is a difference in the entropy between liquid and solid states), we calculated the driving force for crystallization. The last item necessary for calculation of the overall crystallization rate physical quantity is the diffusion constant. At this point, it must be noted that the time scales accessible in standard computational experiments are too short to obtain data for the deep supercooled state. Therefore, the *D*(*T*) dependences obtained at analyzed isobaric conditions have been fitted by the well-known Vogel–Fulcher–Tammann (VFT) equation, which enables approximation of the diffusion constant at low temperatures. The simulations *D* estimated from the molecular dynamics are presented in the inset of Figure 7. Consistent with previous reports, the dynamics of *S*- and *RS*-ibuprofen are similar.^22,68^ Taking this fact into account, we decided to fit the same VFT equation for both materials at a given pressure. Additionally, comparison between the results of our dielectric measurement performed for *S*- (see Supporting Information) and those published for *RS*-ibuprofen^25,38^ revealed that both systems exhibit a very similar temperature dependence of the structural relaxation time at constant ambient and elevated pressure.^22^ Finally, we estimated the overall crystallization rate *k* using eqs 3, 4, and 5. The results are shown in the upper panel of Figure 7.

One can observe that the combination of CNT and the normal model of growth does not accurately predict the experimental *k* values for examined substances. However, we would like to stress the crucial difference between the procedure of the experiment and the simulation CNT. The theory predicts how the liquid crystallizes during the cooling. This concept assumes that the two components of the overall crystallization process, i.e., the nucleation and crystal growth, occur at different temperature ranges. The curve which represents crystal growth is located close to the melting temperature, whereas the nucleation is more pronounced at lower temperatures. If both stages are separated, the system does not crystallize because the critical nuclei are formed close to the glass transition, at temperatures at which the crystal growth is suppressed by insufficient molecular mobility. This situation takes place for both *S*- and *RS*-ibuprofen, which during cooling in the experimental study exhibits the tendency to form a glass. Hence, to induce the crystallization and then to study its rate, we led the substances to a temperature a little bit lower than the glass transition temperature. This procedure enables creation of the critical nuclei. Subsequently, we heated the system to the temperature where crystal growth proceeds and investigated the overall crystallization process. Taking this fact into account and analyzing the presented theoretical predictions given in the upper panel of Figure 7, one should notice two crucial observations. First, the computed values of *k* are smaller than the experimental. It is because the experimentally observed crystallization rates are obtained for the crystallization process which is stimulated by the experiment procedure, i.e., initiation of the nucleation rate at low temperatures. The studied substances do not crystallize during the applied cooling, and therefore one might expect that the experimental *k* should be indeed smaller for the cooling experiment. Second, the experimentally recorded crystallization of *S*- and *RS*-ibuprofen is governed by the crystal growth rate, and therefore it is detected closer to the melting temperature than the theoretical expectations. In the lower panel of Figure 7 we present both *N* and *U*, and one can clearly see that the shapes of the experimental *k* curves correspond well to the theoretical *U* curves. Moreover, the experimentally determined difference between overall crystallization rates at *p* = 0 MPa and *p* = 200 MPa for *RS*- and *S*-ibuprofen correspond to behaviors of the crystal growth rates. In this context, it is worth noting that CNT predicts the increase in the pressure causes the decrease in the nucleation rate for both materials. However, the overall crystallization rate behaves differently for *RS*- and *S*-ibuprofen, which is probably the effect of the increase in the crystal growth rate. At given *T*_m_ – *T*, the gain in *U* compensates for the slowdown of the nucleation, which in the case of *RS*-ibuprofen results in the theoretically predicted increase in the crystallization tendency.

At this point, we should also comment on the fact that on the basis of the result presented in Figure 1 one can conclude that *RS*-ibuprofen exhibits a higher crystallization tendency (the racemic mixture crystallizes during the heating), whereas *k* values presented in upper panel of Figure 7 are smaller for this substance. Typically, it is considered that both quantities are coupled, and consequently the theoretically predicted *k* should be higher for *RS*-ibuprofen. However, our results revealed differences, which might be due to different experimental procedures. As we already mentioned, in studied cases, i.e., when crystallization is triggered by prior cooling of the system up to *T*_g_, the overall crystallization rate is mainly governed by *U*. In this context, it is worth noting that in Figure 1*RS*-ibuprofen crystallizes about 40 K below *T*_m_. Interestingly, at corresponding temperatures, i.e., at *T* > *T*_m_ – 40, *U* for *RS*-ibuprofen is higher than for pure *S*-enantiomer; see lower panel of Figure 7. Hence, we can suspect that during calorimetric experiments *U* for *S*-ibuprofen is not sufficient to progress the crystallization of the system, especially during the applied heating rate. It is because during fast heating the nucleation is strongly limited, and consequently the role of crystal growth in crystallization increases. However, when the heating from glass to desire temperature is slower, like took place during dielectric measurements, the role of nucleation is less suppressed. Consequently, participation of *N*, which exhibits higher values for *S*-ibuprofen, in the overall crystallization process becomes more effective, and then we observe the higher *k* values for *S*-ibuprofen. Hence, the presented results put attention on the interesting aspect of the crystallization studies, which is the connection between the heating rate and the system stability behavior.

**Figure 8:**
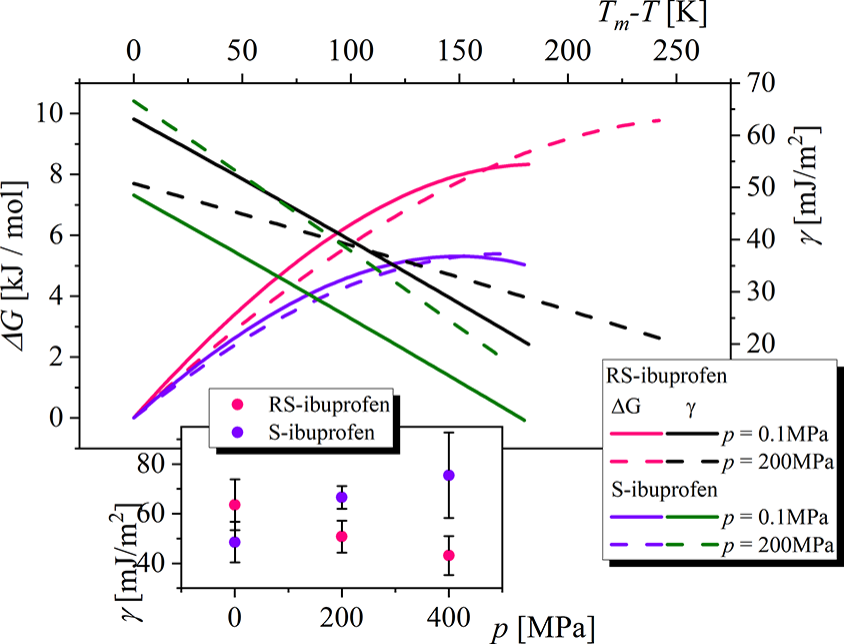
Temperature dependence of the driving force for the crystallization process and the solid–liquid interfacial free energy calculated for *RS*- and *S*-ibuprofen at ambient and elevated pressure. The inset presents the value of the solid–liquid interfacial free energy as a function of pressure.

Nevertheless, combining upper and lower panels of Figure 7, we can state that the combination of CNT and the normal model of growth qualitatively predicts differences in the rate of the crystallization process, which are experimentally observed for *RS*- and *S*-ibuprofen; i.e., at ambient pressure, *S*-ibuprofen crystallizes faster than the racemic mixture. Hence, CNT could be used to investigate the reason for the faster crystallization of pure *S*-ibuprofen. In this context, we would like to recall that the dynamics of the two studied systems are almost identical. Therefore, thermodynamic aspects of the crystallization process should be examined in detail.^22,68^ Consequently, in Figure 8 we present Δ*G* and γ for both substances.

As one can observe, at ambient pressure, Δ*G* and γ are higher for *RS*-ibuprofen than for the pure S-enantiomer at all temperatures. On the basis of the expression of the nucleation barrier ΔG* (see eq 4), a higher driving force Δ*G* facilitates nucleation. Oppositely, a higher γ makes the creation of the solid–liquid interface more costly in free energy and so does not favor nucleation. It thus seems that the contribution of γ dominates in the crystallization trend between *RS*- and *S*-ibuprofen. The mutual relationship between both discussed quantities results in the smaller values of the nucleation barrier () for *S*-ibuprofen (results not presented). During compression, both materials present similar trends for Δ*G*; see Figure 8. Interestingly, as we present in the inset of Figure 8, the pressure dependence of γ_m_ and thus γ (see eq 7) is entirely different for two materials; i.e., the increase in the pressure implies an increase in the γ_m_ and then an impediment of the crystallization for *S*-ibuprofen, whereas for racemic mixture γ_m_ decreases during compression. To confirm this observation, we used the CFM to calculate γ_m_ at 400 MPa; see inset in Figure 8. The obtained results are in agreement with the trend recorded experimentally from 0 to 200 MPa; i.e., when pressure increases, the crystallization process slows down more significantly in the case of the S-enantiomer. Hence, we can state that the γ is the main factor leading to observed differences in the crystallization rates for *S*- and *RS*-ibuprofen. In this context, it has to be stressed that, in the presented study, we have not been able to experimentally examine the crystallization process kinetics at 400 MPa due to the limits of the equipment used.

The fact that γ_m_ for *RS*-ibuprofen is higher than that for pure S-enantiomer could be naturally explained by the fact that both materials crystallize to different space groups, i.e., *P*2_1_/*c* for *RS*-ibuprofen and *P*2_1_ for *S*-ibuprofen.^24^ Thus, naturally we might expect different values of the solid–liquid interfacial free energy at given melting conditions. Moreover, their temperature–pressure dependences of γ_m_ would also be different. Nevertheless, it must be noted that in most cases the racemic solid compounds are often marginally stable over the enantiopure forms.^69,70^ This trend is well in line with the density of the crystal form of *RS*-ibuprofen ρ^*RS*^ = 1.12 g/cm^3^,^71^ which is slightly above the density of *S*-ibuprofen ρ^*S*^ = 1.09 g/cm^3^.^72^ Assuming that the liquid forms of *RS*- and *S*-ibuprofen are similar (they possess similar mobility, *T*_g_, etc.), one can thus expect that the more ordered crystalline *RS*-ibuprofen form shows a larger difference with its liquid state than the *S*-ibuprofen, and consequently γ_m_^*RS*^ > γ_m_^*S*^. Additionally, it is worth noting that the liquid of *RS*-ibuprofen is intrinsically more disordered than *S*-ibuprofen because of the R and S mixture. This enantiomeric disorder does not seem to impact the dynamics; however, there is a difference in the entropy between RS and S, which as well means that γ_m_^*RS*^ > γ_m_^*S*^. We would like to also mention that recent studies pointed out that *R*- and *S*-ibuprofen could persist in anti- as well as syn-periplanar conformation.^24^ The latter implies that to form the solid–liquid interface, the molecules have to undergo anti- to syn-transition because crystal structures of *S*- and *RS*-ibuprofen are comprised solely from molecules exhibiting syn-periplanar conformation. Hence, the interface’s formation requires additional work related to the transition from anti- to syn- conformation. Importantly, the recent results report that at ambient pressure and at a given temperature, the number of molecules characterized by the antiperiplanar conformation is higher for the racemic mixture.^24^ Consequently, the energy needed to form the interface is higher, and therefore one can expect the higher value of γ_m_ for *RS*-ibuprofen.

In the case of the pressure dependence of γ_m_, it should be recalled that despite its importance in the physical description of the crystal morphology or the nucleation rate, only a few experimental data of γ are actually available in the literature and mostly for metallic alloys. Data for molecular compounds are even scarcer.^73−75^ To the authors’ knowledge, no experimental data on the pressure dependence of the solid–liquid interfacial energy exist, and only a few numerical works have been performed mostly on Lennard-Jones simple atomic systems and water.^76−78^ From these simulations, it seems that the solid–liquid interfacial free energy γ_m_ increases along the coexistence line; i.e., γ_m_ increases upon increasing pressure or temperature as observed for *S*-ibuprofen in the present study. Nevertheless, to examine in detail and then to understand the effect of the compression on the solid–liquid interfacial free energy, additional work has to be performed.

Finally, it seems to be interesting to compare the obtained values of γ_m_ for *S*- and *RS*-ibuprofen with some other values reported for pharmaceutical substances such as nifedipine (21.5 and 14.4 mJ/m^2^ for α and β polymorphic form respectively), felodipine (28.7 and 15.5 for I and II form respectively), and indomethacin (22 and 27 mJ/m^2^ for I_α_ and II_γ_ form respectively). One can notice that both forms of ibuprofen are characterized by values of γ_m_, which are about two times higher than for other mentioned substances. It implies that the nucleation rate of ibuprofen would be expected to be relatively low, and hence vitrification of this drug is facilitated.

Summarizing, in this paper, we examined in detail the crystallization process for the S-enantiomer and the racemic mixture of ibuprofen at ambient and elevated pressures. Experimental studies revealed that the S-enantiomer crystallizes faster than the racemic mixture at ambient pressure conditions. Employing the CNT and the normal model of the crystal growth, we predict that the increase in the pressure means that the crystallization of the S-enantiomer slows down, whereas it accelerates for a racemic mixture. Taking into account that both studied systems are characterized by almost identical molecular mobility, the thermodynamic aspect of the crystallization process should be responsible for the observed differences in the crystallization rate. The results of the performed computational studies show that at ambient pressure γ_m_ for *S*-ibuprofen is smaller than for the racemic mixture. Additionally, we present that when the pressure increases γ_m_ behaves entirely differently for *S*- and *RS*-ibuprofen. In the case of the racemic mixture γ_m_ decreases, whereas for the S-enantiomer it increases, which corresponds to the behavior of the overall crystallization rate. Hence, taking into account that the diffusion and driving force, i.e., the other factors influencing the rate of the formation of the nuclei within the liquid, exhibit very similar changes during compression, the solid–liquid interfacial free energy variations should be considered as the main factor responsible for the theoretically predicted behavior of the crystallization process. This observation seems to be even more intriguing if one considers that γ_m_ hypothetically might be sensitive not only for the crystal structure formed but also for the composition of different conformations within the liquid system. The latter implies that the appropriate molecular composition might be used for the practical management of the crystallization process. Hence, from the presented study, we suggest new ways leading to the control of the stability behavior of materials below the melting temperature, which is extremely advantageous in the case of poorly water-soluble and therefore slightly bioavailable crystalline pharmaceuticals.

## Supporting Information Available

Description of the dielectric data analysis leading to the determination of the glass transition temperatures and the isobaric fragility (PDF)
