# Supplementary material for: Pressure Dependence of the Crystallization Rate for the S-Enantiomer and a Racemic Mixture of Ibuprofen
Source: Cryst Growth Des. 2021 Oct 27;21(12):7075–86. doi: 10.1021/acs.cgd.1c00980 (PMC8641391; doi:10.1021/acs.cgd.1c00980)
Supplement: Supplementary file 1 — cg1c00980_si_001.pdf [file cg1c00980_si_001.pdf]

The pressure dependence of crystallization rate for S-enantiomer and racemic mixture of ibuprofen

Kajetan Koperwas[1,2], Wenkang Tu[1,2], Frédéric Affouard[3], Karolina Adrjanowicz[1,2], Filip Kaskosz[1,2], and Marian Paluch[1,2]

1. University of Silesia in Katowice, Institute of Physics, 75 Pułku Piechoty 1, 41-500 Chorzów, Poland
2. Silesian Center for Education and Interdisciplinary Research SMCEBI, 75 Pułku Piechoty 1a, 41-500 Chorzów, Poland
3. Univ. Lille, CNRS, INRAE, Centrale Lille, UMR 8207 - UMET - Unité Matériaux et Transformations, F-59000 Lille, France

* Corresponding author: kajetan.koperwas@us.edu.pl

Supporting Information

Dielectric Spectroscopy

As seen in Figure S1, we performed dielectric measurements by varying the temperature along various isobars, namely p= 0.1 MPa, 200 MPa and 400 MPa, and also by varying the pressure along an isotherm of T= 254.2 K. To analyze the results, we fitted the structural α- relaxation peak visible in each dielectric loss ε'' spectrum by applying the empirical Havriliak – Negami function (HN),[5]

$$\varepsilon^*_{HN}(\omega) = \varepsilon'(\omega) - i\varepsilon''(\omega) = \varepsilon_\infty + \frac{\Delta\varepsilon}{[1+(i\omega\tau_{HN})^a]^b} \quad (1)$$

where $\varepsilon'(\omega)$ and $\varepsilon''(\omega)$ are the real and imaginary parts of complex permittivity, respectively; ε_∞ , $\Delta\varepsilon$ and τ_{HN} represent the high frequency dielectric constant, dielectric strength and relaxation time of a relaxation peak, respectively; a and b denote the profile shape factors of the

relaxation dispersion. Exemplified fitting results to the ε'' spectra obtained at T= 254.2 K, p= 10 MPa and at T= 294.9 K, p= 200 MPa are shown in panels (b) and (c) of Figure S1. Based on the fitting parameters, we can calculate the structural α- relaxation time, τ_α, in terms of the following equation,[6]

$$\tau_\alpha = \tau_{HN}[\sin(\frac{\pi a}{2+2b})]^{-1/a} * [\sin(\frac{\pi ab}{2+2b})]^{1/a} \quad (2)$$

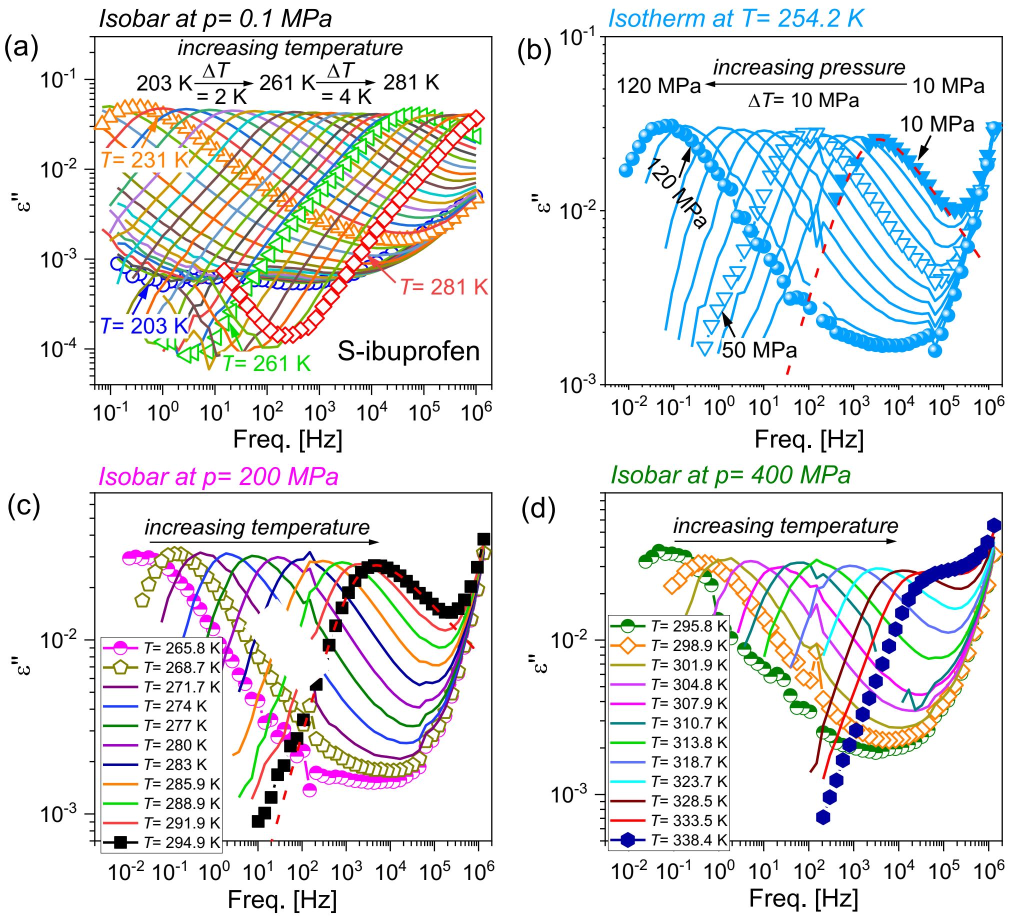

Figure S1. Isobaric measurements performed at pressures of p= 0.1 MPa (a), 200 MPa (c) and 400 MPa (d). Isothermal measurements performed at the temperature of T= 254.2 K (b). Exemplified fitting results to the structural α- relaxation peaks by using the empirical HN equation are shown in panels (b) and (c) as red dashed lines.

In Figure S2a, we established the reciprocal temperature dependences of τ_α ascertained in isobaric measurements. Meanwhile, the pressure dependence of τ_α ascertained in the isothermal

measurement is depicted in Figure S2b. To describe such temperature and pressure dependences of τ_α, we employed the Vogel – Fulcher- Tamman (VFT) equation and its pressure counterpart,[1,2]

$$log_{10}\tau_\alpha = log_{10}\tau_0 + \frac{DT_0}{T-T_0} \quad (3)$$

$$log_{10}\tau_\alpha = log_{10}\tau_0 + \frac{D_p P}{P_0-P} \quad (4)$$

where τ_0, D, T_0, D_p and P_0 are fitting parameters. The glass transition temperature T_g/glass transition pressure P_g can be counted as the temperature/pressure at which τ_α equals to 100 s. In addition, in Figure S2a, another significant parameter, the fragility index m_p, can be determined,[7]

$$m_p = \left.\frac{\partial \log_{10}\tau_\alpha}{\partial(T_g/T)}\right|_{p=const,T=T_g} \quad (5)$$

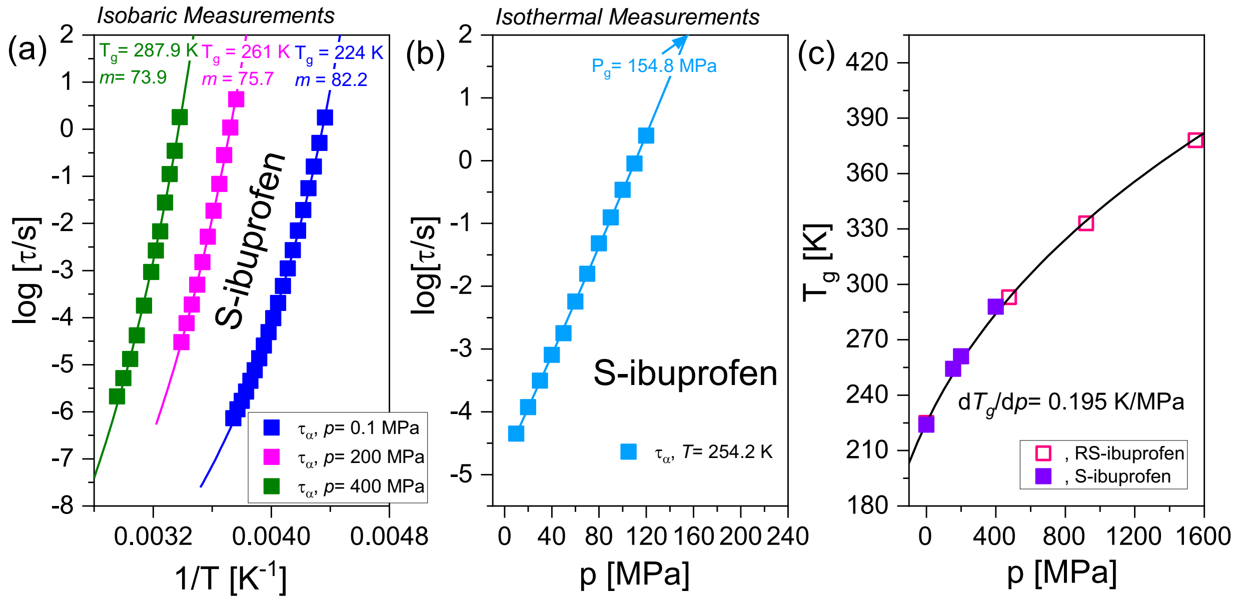

Figure S2. Temperature dependences of structural relaxation times τ_α obtained for S-ibuprofen from various isobaric (a) and isothermal (b) measurements. Solid lines represent the fits in terms of the Vogel-Fulcher-Tamman (VFT) equation and its pressure counterpart.[1,2] Panel (c): Dependence of the glass transition temperatures $T_g s$ for S-ibuprofen as a function of pressure. The data for RS- ibuprofen taken from our previous work[3] are added for comparison. Black solid line represents the fitting result by using the Andersson – Andersson equation.[4]

The m_p values are 82.2, 75.7 and 73.9 for S-ibuprofen at pressures of *p*= 0.1 MPa, 200 MPa and 400 MPa, respectively. It is apparent that m_p decreases as the increasing pressure. Similarly, as reported for RS- ibuprofen in our previous work, the m_p value is 87 $\pm$2 at *p*= 0.1 MPa while 72.5 $\pm$3.5 at *p*= 920 MPa.[3]

Based on the ascertained T_g and P_g results for S-ibuprofen, we prepared the plot of T_g as a function of pressure in Figure S2c. Moreover, data for RS- ibuprofen as reported in our previous work are added for comparison.[3] It is notable that data for S- ibuprofen and RS- ibuprofen are identical. In order to analyze the T_g - p dependences, we applied the Andersson – Andersson equation,[4]

$$T_g(P) = T_g(0)(1 + \frac{b}{c}p)^{1/b} \tag{6}$$

where $T_g(0)$, *b* and *c* are fitting parameters. The same fitting results are obtained for S- ibuprofen and RS -ibuprofen. Moreover, in the limit of zero pressure, the same dT_g/dp result of 0.195 K/MPa is determined for both chemicals.

Molecular Dynamics Simulations

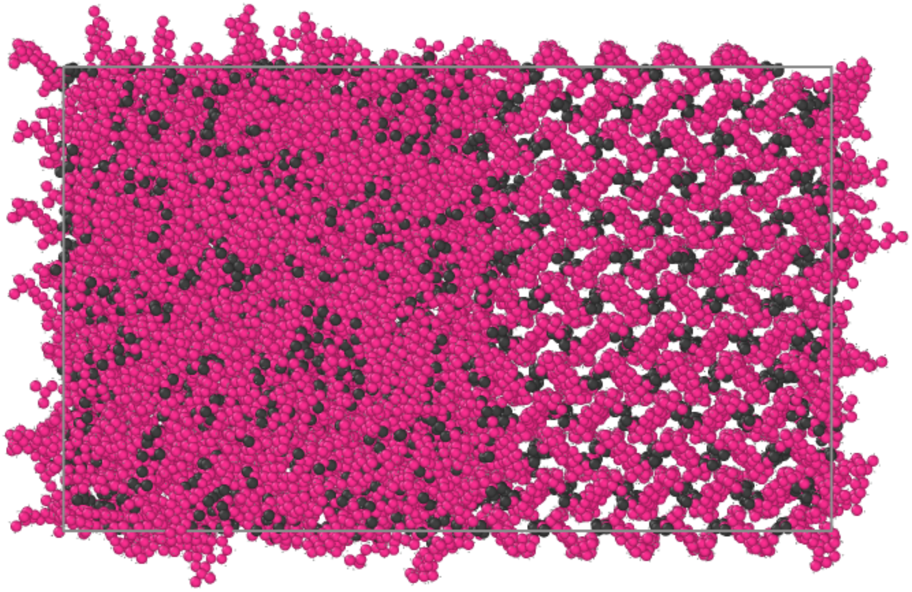

Figure S3. The final configuration of the biphasic system, which simulation enables confirmation of utility of the experimentally determined melting temperatures for further computational experiments. As one can observe any undesired crystallization or melting event are not detected.

REFERENCES:

1. Paluch, M., Rzoska, S. J., Habdas, P. & Ziolo, J. Isothermal and high-pressure studies of dielectric relaxation in supercooled glycerol. *J. Phys. Condens. Matter* **8**, 10885–10890 (1996).
2. Paluch, M., Ziolo, J., Rzoska, S. J. & Habdas, P. The influence of pressure on dielectric relaxation for phthalate derivatives in the supercooled state. *J. Phys. Condens. Matter* **9**, 5485–5494 (1997).
3. Adrjanowicz, K. *et al.* Dielectric relaxation and crystallization kinetics of ibuprofen at ambient and elevated pressure. *J. Phys. Chem. B* **114**, 6579–6593 (2010).
4. Andersson, S. P. & Andersson, O. Relaxation Studies of Poly(propylene glycol) under High Pressure. *Macromolecules* **31**, 2999–3006 (1998).
5. Havriliak, S. & Negami, S. A complex plane representation of dielectric and mechanical relaxation processes in some polymers. *Polymer (Guildf).* **8**, 161–210 (1967).
6. Knapik-Kowalczuk, J., Tu, W., Chmiel, K., Rams-Baron, M. & Paluch, M. Co-Stabilization of Amorphous Pharmaceuticals—The Case of Nifedipine and Nimodipine. *Mol. Pharm.* **15**, 2455–2465 (2018).
7. Angell, C. . Relaxation in liquids, polymers and plastic crystals — strong/fragile patterns and problems. *J. Non. Cryst. Solids* **131**–**133**, 13–31 (1991).
